# Design of Functionalized Silica Immobilization of PETase from *Kibdelosporangium aridum*: Comparison of Glyoxyl and Glutaraldehyde Strategies for PET Depolymerization

**DOI:** 10.1021/acsomega.6c00584

**Published:** 2026-04-20

**Authors:** Buse Çaloğlu Susamaz, Mine Nazan Kerimak-Öner, N.Ece Varan Faki, Leyla Colakerol Arslan, Deniz Yildirim, Barış Binay

**Affiliations:** † Gebze Technical University, Faculty of Engineering, Department of Bioengineering, Gebze, Kocaeli 41400, Türkiye; ‡ Kocaeli University, İzmit Vocational School, Department of Medicinal and Aromatic Plants, Kartepe, Kocaeli 41285, Türkiye; § 37506 Cukurova University, Faculty of Science and Letters, Department of Chemistry, Adana 01330, Türkiye; ∥ 52962 Gebze Technical University, Faculty of Science, Department of Physics, Gebze, Kocaeli 41400, Türkiye; ⊥ Cukurova University, Faculty of Engineering, Department of Chemical Engineering, Adana 01330, Türkiye; # BAUZYME Biotechnology Co., Gebze Technical University, Technopark Region, Gebze, Kocaeli 41400, Türkiye

## Abstract

Polyethylene terephthalate (PET) is a widely used thermoplastic that poses a major challenge to global resource sustainability because of its extensive consumption and contribution to plastic pollution. This study aimed to enhance the performance of PETase derived from *Kibdelosporangium aridum* (*Ka*PETase) through immobilization onto aldehyde-functionalized silica supports with varying active groups: (i) 3-aminopropyl silica gel via glutaraldehyde (Si-NH_2_@*Ka*PETase), (ii) (3-aminopropyl)­triethoxysilane (3-APTES) functionalized silica via glutaraldehyde (Si-Glu@*Ka*PETase), and (iii) glyoxyl silica (Si-Ald@*Ka*PETase). The immobilized biocatalysts exhibited 4.6- to 5.1-fold greater thermal stability at 75 °C than that of the free enzyme. Catalytic efficiency was also significantly enhanced, increasing by 2.6- to 5.0-fold. Under optimized conditions, PET depolymerization assays demonstrated improved hydrolytic performance. HPLC analysis confirmed terephthalic acid (TPA) and mono­(2-hydroxyethyl) terephthalate (MHET) as the primary degradation products. After 1 h of reaction, degradation product concentrations reached 43.4, 75.6, 93.4, and 61.2 mg mg^–1^ protein for free *Ka*PETase, Si-NH_2_@*Ka*PETase, Si-Glu@*Ka*PETase, and Si-Ald@*Ka*PETase, respectively. Surface-sensitive XPS measurements revealed more pronounced PET surface chemical modifications for PET surfaces incubated by aldehyde- and especially glutaraldehyde-functionalized *Ka*PETase systems, indicating enhanced enzyme–surface interactions. Overall, immobilization on functionalized silica supports significantly improved catalytic activity, thermostability, and reusability. These findings demonstrate the strong potential of robust immobilized *Ka*PETase systems for sustainable and industrially relevant PET biodegradation.

## Graphical abstract

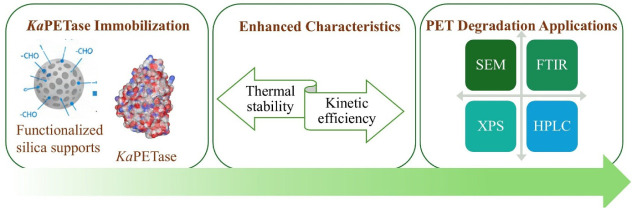

## 1 Introduction

The rapid expansion of the global population has driven increased consumption of high-demand synthetic polymeric materials in daily use products. PET stands out as a highly prominent polymer owing to its favorable physicochemical properties, which facilitate its widespread application across diverse sectors, including packaging, textiles, automotive components, medical devices, and electronics.^1^ PET is a linear polyester synthesized through the polycondensation of two cornerstone polymer precursors, TPA and ethylene glycol (EG), yielding a backbone characterized by repeating ester linkages.^2^ Since its initial development by DuPont in the 1940s^3^, PET has become one of the most heavily manufactured synthetic polymers, with global production surpassing 55 million tons in 2021. The market is further projected to reach approximately USD 41 billion by 2030, reflecting a compound annual growth rate (CAGR) of 10%.^4^ Despite its utility, PET’s remarkable resistance to both abiotic and biotic degradation enables its persistence in terrestrial and marine environments for up to 500 years. Therefore, the case has been leading to substantial plastic accumulation and long-term environmental contamination^5,6^ and necessitates the development of innovative and highly effective polymer degradation strategies. Among the current PET decomposition approaches, enzyme-mediated biocatalysis offers a particularly promising and sustainable alternative. This method provides an environmentally end-of-life route that efficiently recovers and enables the reuse of the polymeric constituents.^7–10^

PHEs belong primarily to the hydrolase class, encompassing and include esterases, lipases, and cutinases, all of which are capable of initiating the enzymatic depolymerization of PET. Notably, PET-degrading cutinases (often simply termed PETases) catalyze the cleavage of ester bonds, a reaction naturally employed to break down the long-chain fatty acids in plant cutin polymer.^2^ Given that the PET backbone is defined by repeating ester linkages, it presents an analogous synthetic substrate for these enzymes.^6^ However, a major challenge in PET biocatalysis stems from the highly crystalline structure of conventional PET materials, which severely limits enzyme accessibility to hydrolyzable bonds. To overcome this limitation and enhance the amorphous fraction of the polymer, pretreatment methods are essential. Thermal extrusion is a common technique used to increase substrate bioavailability, which involves heating the PET above its glass transition temperature (*T*_g_). This critical temperature is typically reported to be 75–80 °C in air and 65–70 °C under aqueous reaction conditions.^10,11^ The requirement for this high reaction temperature underscores the need for thermally robust biocatalysts in industrial PET degradation processes.

A subset of PHEs, isolated from thermophilic bacterial species such as *Thermobifida fusca*,^12^ *Thermobifida alba*,^13^ and *Thermofida cellulosilytic* ^14^ exhibit enhanced activity at elevated temperatures. These enzymes are critical, given the thermal requirements for increasing the substrate bioavailability of crystalline PET. Nevertheless, even among these thermostable variants, only a select few possess the requisite thermal robustness and reusability demanded for sustained operation within industrial-scale PET recycling processes.^15^ Despite their inherent catalytic promise, the broad industrial implementation of PHEs remains severely constrained by limitations, including inadequate operational stability, poor reusability, and diminished specific activity under the harsh temperatures and chemical environments characteristic of polymer processing. Consequently, strategies to augment the intrinsic stability and functional longevity of these biocatalysts are essential for advancing industrial PET depolymerization.

Enzyme immobilization is widely used to improve enzyme reusability, operational stability, and process efficiency in biocatalytic applications. However, immobilization does not necessarily guarantee improved catalytic performance, as the final properties of the immobilized enzyme strongly depend on the immobilization protocol, the support properties, and the structural characteristics of the enzyme. Immobilization may alter enzyme activity, selectivity, and stability due to structural constraints or changes in the microenvironment surrounding the enzyme. In particular, multipoint covalent immobilization has been widely reported as an effective strategy to enhance enzyme rigidity and thermal stability by forming several covalent linkages between the enzyme surface and the support. Nevertheless, inappropriate immobilization strategies may lead to activity loss or diffusion limitations. Therefore, careful design of immobilization strategies remains essential. Recent discussions have also addressed whether enzyme immobilization can be considered a mature discipline, highlighting that despite decades of research, new materials and immobilization strategies continue to emerge for improving biocatalyst performance.^16–22^ In particular, immobilization techniques allow the operational limitations of free PHEs to be overcome. Collectively, the indicated critical advantages significantly expand the feasibility of deploying PHEs in large-scale plastic bioconversion systems.^15^ Despite the clear benefits, the investigation into PHE immobilization on solid supports remains relatively limited, with reported materials including Co_3_(PO_4_)_2_^23^, ZIF-8^24^, Fe_3_O_4_,^25^ magnetic biochar^26^ mesoporous silica,^27^ and silica (SiO_2_).^28^ Among these, silica is an especially attractive support. Its appeal is rooted in its inherent chemical and thermal stability, high surface area, and the convenience of surface functionalization with diverse chemical groups to ensure effective and secure enzyme attachment. Consequently, functionalized silica materials have been extensively investigated and validated as immobilization platforms for a wide range of enzyme classes, demonstrating improvements in catalytic activity and thermostability, and thereby contributing significantly to both the scientific literature and industrial applications.^16,29–31^ Furthermore, functionalized silica supports address the dual challenge of preserving active site integrity while enhancing conformational rigidity. By shifting from nonspecific lysine-based attachment toward site-specific, multipoint covalent immobilization on robust silica matrices, researchers can develop recyclable biocatalysts that exhibit improved stability and catalytic performance compared to their native counterparts under diverse industrial conditions.^32–34^

The characteristics of the active group, such as the functional group involved in binding, chain length, flexibility, and hydrophilic–hydrophobic balance, play a decisive role in determining the catalytic behavior of covalently immobilized enzymes. These structural parameters influence enzyme orientation, conformational mobility, accessibility of the active site, and resistance to denaturation.^15,35,36^ Although a broad range of functionalized carriers has been developed for immobilization, aldehyde-containing supports remain among the most frequently employed due to their strong reactivity toward nucleophilic residues on protein surfaces and their ability to promote multipoint attachment, thereby enhancing structural stabilization.^16,19^

Glutaraldehyde-activated supports are widely used in enzyme immobilization due to their ability to generate reactive aldehyde groups that can interact with amino groups on enzyme surfaces.^37,38^ However, the immobilization mechanism on these supports may involve a combination of ionic adsorption and covalent interactions depending on the immobilization conditions and the structure of the support.^39^ In addition, glutaraldehyde may form polymeric structures or cyclic intermediates on aminated supports, generating heterogeneous reactive surfaces that influence enzyme orientation and immobilization efficiency. Recent studies have emphasized the importance of understanding these heterofunctional properties when interpreting immobilization mechanisms on glutaraldehyde-modified supports.^40,41^

Glyoxyl-activated supports represent a well-established strategy for multipoint attachment of enzymes. These supports contain aldehyde groups that react preferentially with deprotonated ε-amino groups of lysine residues, requiring immobilization under alkaline conditions (typically around pH 10). At this pH, nucleophilic attack by lysine residues is favored, enabling the formation of multipoint attachment between the enzyme surface and the support.^19,42^ This multipoint attachment often results in significant structural rigidification, leading to marked improvements in thermal stability, operational stability, and resistance to organic solvents. Furthermore, the initial interaction between the enzyme and the support is generally weak, thereby reducing nonspecific adsorption and promoting intense multipoint covalent immobilization.^43^ However, glyoxyl supports present several limitations. Alkaline conditions required for immobilization may compromise enzyme stability, particularly in pH-sensitive proteins, potentially causing partial inactivation. Additionally, the covalent bonds initially formed (Schiff bases) must be stabilized by reduction (e.g., with sodium borohydride) to prevent reversibility, which introduces an additional chemical step that may affect enzyme structure.^44^

The present study details a targeted immobilization strategy using functionalized silica supports for the recently characterized PETase from *Kibdelosporangium aridum* (*Ka*PETase, UniProt ID: A0A1W2FU08). *Ka*PETase is a monomeric including 263 amino acids without signal sequence, approximately 33 kDa α/β-hydrolase-fold enzyme. This enzyme is of particular industrial interest because its thermal stability enhances its applicability under operationally demanding conditions. Our primary aim was to comprehensively assess the efficacy of the immobilization approach. To this end, the immobilized biocatalysts underwent extensive physicochemical characterization using Fourier-transform infrared spectroscopy (FTIR), scanning electron microscopy (SEM), and thermogravimetric analysis (TGA). Furthermore, the biochemical and kinetic properties of the immobilized enzyme preparations were systematically evaluated and compared with those of the free *Ka*PETase to quantify improvements in catalytic efficiency, thermal and operational stability, and industrial reusability. Finally, the PET-degrading potential of each immobilized preparation was rigorously examined through controlled incubation with PET substrates, and degradation products and residual materials were characterized using HPLC, XPS, SEM, and FTIR.

## 2 Experimental Methods

### 2.1 Microorganisms, Chemicals, and Kits

Unless otherwise specified, all commercial kits were procured from Thermo Fisher Scientific (Waltham, MA, USA). 3-Aminopropyl-functionalized silica gel (Si-NH_2_, 40–63 μm, ∼1 mmol/g NH_2_ loading), silica, (3-aminopropyl)­triethoxysilane (3-APTES, ≥98%), glycidol (96%), sodium metaperiodate (≥99.0%), and sodium borohydride (99.0%) were bought from Sigma-Aldrich (St. Louis, MO, USA). The recombinant *E. coli* BL21 (DE3) strain, utilized for the expression of *Ka*PETase, was sourced from the GERC laboratory. The autoinduction medium necessary for protein expression was purchased from Formedium (Swaffham, Norfolk, UK). Finally, Nickel–nitrilotriacetic acid (Ni-NTA) HisTrap columns and PD-10 desalting columns were obtained from Cytiva (Marlborough, MA, USA).

### 2.2 Expression and Purification of *Ka*PETase

The heterologous expression and purification of *Ka*PETase from recombinant *E. coli* BL21 (DE3) were conducted following the detailed procedure established in previous work.^24^ During the affinity chromatography step, purified *Ka*PETase was eluted using a buffer containing 200 mM imidazole. The collected fractions were concentrated using ultrafiltration membranes with a 10-kDa molecular weight cutoff (MWCO). Subsequently, the concentrated samples were purified using a PD-10 desalting column to facilitate buffer exchange. The resulting enzyme fractions were immediately aliquoted and subsequently stored at −80 °C. This storage regimen was strictly followed to maintain enzyme stability and prevent activity loss due to repeated freeze–thaw cycles.

### 2.3 Preparations and Activations of Silica Supports

Three different functionalized silica supports were prepared for *Ka*PETase immobilization: aminated silica (Si-NH_2_), glutaraldehyde-activated aminated silica (Si-Glu), and glyoxyl-functionalized silica (Si-Ald).

#### 2.3.1 Preparation of Glutaraldehyde-Activated Silica (Si-NH_2_ and Si-Glu)

Commercial 3-aminopropyl-functionalized silica (Si-NH_2_) was used as the starting support. For activation with glutaraldehyde, 1 g of Si-NH_2_ was suspended in 25 mL of sodium phosphate buffer (25 mM, pH 7.0) containing 1.25% (w/w) glutaraldehyde and gently agitated at 25 °C for 2 h. After activation, the support was collected by filtration and extensively washed with distilled water to remove unreacted glutaraldehyde. The activated support was then dried at room temperature.

To prepare glutaraldehyde-modified silica with a longer linker structure (Si-Glu), silica particles (Si–OH) were first functionalized with amino groups. Briefly, 1 g of silica was suspended in 25 mL of acetone containing 4% (w/v) 3-APTES and incubated for 12 h under continuous stirring. The aminated silica was then washed thoroughly with distilled water and dried at 60 °C. Subsequently, 1 g of the aminated silica was treated with 25 mL of sodium phosphate buffer (25 mM, pH 7.0) containing 1.25% (w/w) glutaraldehyde for 2 h at 25 °C. After activation, the support (Si-Glu) was washed with distilled water and dried at room temperature.

Glyoxyl-functionalized silica was prepared through a glycidol-mediated surface modification followed by periodate oxidation. Briefly, 1 g of silica (Si–OH) was suspended in 10 mL of 1.7 M NaOH solution containing 0.34 g of NaBH_4_ under ice-bath conditions. Subsequently, 3.6 mL of glycidol was added dropwise and the mixture was stirred continuously for 24 h. In this step, glycidol reacts with surface silanol groups of silica, introducing vicinal diol groups on the support surface. The resulting material was filtered and thoroughly washed with distilled water to remove residual reagents. To convert the vicinal diol groups into aldehyde functionalities, the modified support was treated with 25 mL of 0.1 M sodium metaperiodate solution for 2 h under gentle stirring at room temperature. Periodate oxidation generated surface glyoxyl groups (−CHO). The resulting glyoxyl-functionalized support (Si-Ald) was then washed extensively with distilled water and dried at room temperature.

### 2.4 Immobilization of *Ka*PETase on Functionalized Silica Supports

*Ka*PETase immobilization was performed using the prepared functionalized supports under mild conditions.^36^ For immobilization on glutaraldehyde-activated supports (Si-NH_2_ and Si-Glu), 1 g of each support was incubated with 4 mL of *Ka*PETase solution (0.1 mg mL^–1^) prepared in sodium phosphate buffer (25 mM, pH 7.0). The mixtures were shaken in an orbital shaker at 25 °C for 2 h. During this process, the enzyme is initially adsorbed onto the support surface, which may facilitate subsequent interactions between aldehyde groups on the support and nucleophilic residues on the enzyme surface. After immobilization, the supports were recovered by vacuum filtration and extensively washed with sodium phosphate buffer (25–200 mM, pH 7.0) to remove unbound or weakly adsorbed enzyme molecules.

For immobilization on glyoxyl-functionalized silica (Si-Ald), 1 g of Si-Ald was incubated with 4 mL of *Ka*PETase solution (0.1 mg mL^–1^) prepared in sodium carbonate buffer (25 mM, pH 10.0) at 25 °C for 2 h. Under these alkaline conditions, nucleophilic lysine residues of the enzyme can react with surface aldehyde groups, enabling multipoint attachment. To stabilize the resulting imine bonds, solid sodium borohydride (NaBH_4_) was added to a final concentration of 1 mg mL^–1^, and the suspension was gently stirred for an additional 30 min at 25 °C.^45^ This reduction step converts reversible imine linkages into more stable secondary amine bonds. Finally, the immobilized enzyme preparations were collected by vacuum filtration and extensively washed with distilled water to remove residual reagents and nonbound enzyme.

The collected wash supernatants were retained for the subsequent determination of protein concentration using the BCA protein assay kit.^46^ Finally, the immobilization yield (IY, %) and the recovered activity (RA, %) were calculated using the formulas described by Boudrant et al.^18^

### 2.5 Biochemical and Kinetic Characterization of Immobilized *Ka*PETases

The influence of pH on the activity of both free and immobilized *Ka*PETases was determined by assaying enzyme activity across a range of buffer systems. These systems included 50 mM sodium phosphate (pH 6.5–7.0) and 50 mM Tris-HCl (pH 7.5–9.0). To evaluate the impact of temperature on enzyme activity, reactions were conducted in 50 mM Tris-HCl buffer (pH 8.0) at temperatures ranging from 50 to 80 °C. For both pH and temperature profiling, relative activities (%) were calculated as the ratio of the activity measured under each condition to the maximum observed activity, which was set to 100%.

The thermal stability of the free and immobilized *Ka*PETase preparations was assessed by incubating the preparations at 75 °C for 24 h. Enzyme activities were monitored periodically at 0, 1, 2, 4, 8, 16, and 24 h. Residual activities were calculated as the ratio of the sample activity at a specific time point to its initial activity. The thermal inactivation constant (*k*_d_), half-life values (*t*_1/2_), and stabilizing factors (SF) for the free and immobilized *Ka*PETase samples were mathematically determined according to the procedures outlined by Alagöz et al.^47^

The apparent kinetic parameters of the free and immobilized *Ka*PETase preparations were determined by assaying their specific activities using varying concentrations of the synthetic substrate p-nitrophenyl acetate (p-NPA). All assays were conducted under the previously determined optimum reaction conditions for each preparation. Michaelis–Menten curves were plotted using GraphPad Prism 9.0 ^48^ to accurately determine the apparent Michaelis constant (K_M_) and the maximum reaction rate (*V*_max_) for each biocatalyst.

The operational reuse stability of the immobilized *Ka*PETase preparations was assessed using a series of batch hydrolysis cycles. For each cycle, 50 mg of the immobilized enzyme was incubated with 5 mL of a 1 mM p-NPA solution (final concentration) at 75 °C. Following a short 2 min reaction time, the suspension was centrifuged (1 min at 5000*g*, 4 °C), and the concentration of the hydrolysis product (p-nitrophenol) in the supernatant was quantified by measuring the absorbance at 405 nm. After each cycle, the biocatalysts were carefully washed with 25 mM sodium phosphate buffer (pH 8.0), recovered by filtration, and immediately subjected to a subsequent hydrolysis cycle with a fresh substrate solution. Enzyme activity remaining after each cycle was expressed as a percentage relative to the initial activity measured in the first cycle.

### 2.6 Analytical Methods

#### 2.6.1 *Ka*PETase Activity Test

*Ka*PETase activity was spectrophotometrically determined using p-NPA as a model substrate. The standard reaction mixture consisted of 850 μL of sodium phosphate buffer (50 mM, pH 8.0), and either 50 μL of free *Ka*PETase or 5 mg of immobilized *Ka*PETase. The reaction was initiated by the addition of 100 μL of 5 mM p-NPA dissolved in dimethyl sulfoxide (DMSO) and carried out at 50 °C. The rate of p-nitrophenol (p-NP) production was monitored for 5 min at 405 nm using a UV–visible spectrophotometer (Epoch Spectrophotometer, BioTek).^49^ One unit (U) of *Ka*PETase activity was defined as the amount of enzyme required to catalyze the formation of 1 μmole of p-NP per minute under the specified assay conditions. A control reaction containing buffer but lacking free or immobilized *Ka*PETase, was run in parallel.

#### 2.6.2 Instrumental Analysis of Supports

Morphological characteristics of the immobilization supports, both before and after *Ka*PETase immobilization, were observed using SEM (Philips XL30 SFEG) at magnifications ranging from 2000× to 5000×.^50^ FTIR analysis was performed using the Attenuated Total Reflectance (ATR) technique on all supports, covering a wavenumber range of 500–4000 cm^–1,^ before and after enzyme attachment.^51^ Thermogravimetric analysis (TGA) was performed using a TA Instruments Q500 thermogravimetric analyzer at a heating rate of 10 °C/min from 25 to 800 °C under a nitrogen atmosphere.

### 2.7 Application on Postconsumer PET (PC–PET) Plastic Pieces

PET biodegradation assays were conducted using 0.5 × 0.5 cm pieces of PC–PET, specifically waste PET bottle sections (200 volume) sourced from a local market. These PET pieces were placed into separately into a sodium phosphate buffer (50 mM, pH 8.0) containing immobilized *Ka*PETase preparations. PET pieces were incubated with the immobilized enzyme at a final concentration of 0.072 mg/mL, at 75 °C, for 1 day.

As a control, PET pieces were incubated solely in the buffer solution under identical conditions. Upon completion of the incubation period, all PET pieces were thoroughly rinsed with deionized water (dH_2_O) and dried at 37 °C. After washing step of immobilization support and immobilized enzyme, PET pieces were subsequently analyzed by FTIR, SEM^52^ and XPS.

XPS analysis was conducted under ultrahigh vacuum (UHV) conditions (base pressure <5 × 10^‑10^ mbar) using a SPECS PHOIBOS 150 electron energy analyzer. XPS spectra were acquired at a constant pass energy of 30 eV using an unmonochromated Al Kα X-ray source (1468.6 eV) at a detection angle of 45° with respect to the sample normal. The spectra were calibrated against the Au 4f7/2 peak at 84 eV using an adjacent gold reference sample. Spectral deconvolution was achieved using a mixed Gaussian–Lorentzian fitting function, and background subtraction was performed using the Shirley method. Atomic concentrations were calculated from the integrated intensities following photoionization cross-section corrections.^53^

Additionally, PET pieces were incubated at 75 °C for 60 min, and the formation of TPA was monitored by HPLC at defined time intervals. The degradation products from the *Ka*PETase-catalyzed PET degradation were analyzed using HPLC equipped with a C18 column (Phenomenex Luna, 250 mm × 4.65 mm).^52^ The mobile phase consisted of a mixture of water/acetonitrile (80/20, v/v) containing 0.1% trifluoroacetic acid, delivered at a flow rate of 0.5 mL/min. A 20 μL aliquot of the reaction mixture was diluted with 80 μL of the mobile phase, and the degradation products were detected at 230 nm using a diode array detector. The column temperature was maintained at 30 °C throughout the analysis.

## 3 Results and Discussion

### 3.1 *Ka*PETase Immobilization with Functionalized Silica Support Materials

In this study, silica supports bearing aldehyde functionalities of varying active groups lengths were systematically designed and prepared. As shown in Figure S1, Si-NH_2_ particles were activated with glutaraldehyde molecule to form Schiff base between the primary amino groups on Si-NH_2_ particles and one of aldehyde group of glutaraldehyde molecule. After that, *Ka*PETase was immobilized on the support to form a Schiff base between primary amino groups of *Ka*PETase and the remaining aldehyde group of the glutaraldehyde molecule (Figure S1a). To prepare the Si-Glu support, silica particles were silanized with 3-APTES to generate primary amino groups on the support surface, followed by reaction with glutaraldehyde. Subsequently, *Ka*PETase was immobilized to form a Schiff base (Figure S1b). To prepare Si-Ald support, silica particles were reacted with glycidol to introduce vicinal diol groups on the support, which were subsequently oxidized with periodate to generate glyoxyl (−CHO) groups. After that, *Ka*PETase was immobilized to form Schiff base followed by NaBH_4_ reduction to produce stable secondary amine linkages (Figure S1c). These approaches enabled the preparation of silica supports with varying active groups, allowing the investigation of the influence of aldehyde chain length and surface chemistry on the immobilization efficiency and performance of *Ka*PETase.

The IY (%) and corresponding RA (%) values after immobilization of *Ka*PETase on Si-NH_2_, Si-Glu, and Si-Ald are presented in Table 1.

**Table 1:** Summarization of *Ka*PETase Immobilization on Silica Supports Bearing Different Length of Aldehyde Groups

Support	Added protein amount (mg)	Bound protein amount	IY (%)	RA (%)
Si-NH_2_	0.400	0.384	96	95
Si-Glu		0.344	86	92
Si-Ald		0.360	90	87

The measured IY% and RA% values demonstrated a clear dependence on the chemical nature of the functional groups and on the linker length present on the silica support. Among all carriers tested, the Si-NH_2_ yielded the most favorable results, exhibiting the highest IY (96%) and RA value (95%). These values indicate that Si-NH_2_ provided optimal conditions for covalent attachment while simultaneously preserving the enzyme’s intrinsic catalytic conformation. In contrast, Si-Glu and Si-Ald supports exhibited slightly diminished IY values (86% and 90%, respectively) and RA values (92% and 87%, respectively) when compared to Si-NH_2_. These differences are likely attributable to the chemical characteristics of the aldehyde-based coupling systems and their subsequent influence on enzyme conformation. Specifically, the Si-Glu support employs a longer, bifunctional glutaraldehyde linker that facilitates the formation of multipoint attachments to surface-exposed lysine residues of *Ka*PETase. While this multipoint attachment can enhance the structural rigidity of the enzyme, it may simultaneously impose conformational constraints that lead to the observed reduction in recovered catalytic efficiency. Similarly, Si-Ald promotes Schiff base formation predominantly under alkaline conditions (pH 10.0). This elevated pH may induce minor structural alterations in the *Ka*PETase active site, which could account for the mildly reduced recovered activity observed in this preparation. In addition, the subsequent NaBH_4_ reduction step may further influence enzyme conformation. Although this reduction enhances the stability of the immobilized derivative by preventing bond reversibility, the chemical treatment and exposure to NaBH_4_ can potentially contribute to slight activity losses due to additional structural constraints.^17,54,55^

### 3.2 Instrumental Characterization

SEM analysis was conducted to demonstrate structural changes on the support surfaces after immobilization of the *Ka*PETase (Figure 1).

**Figure 1:**
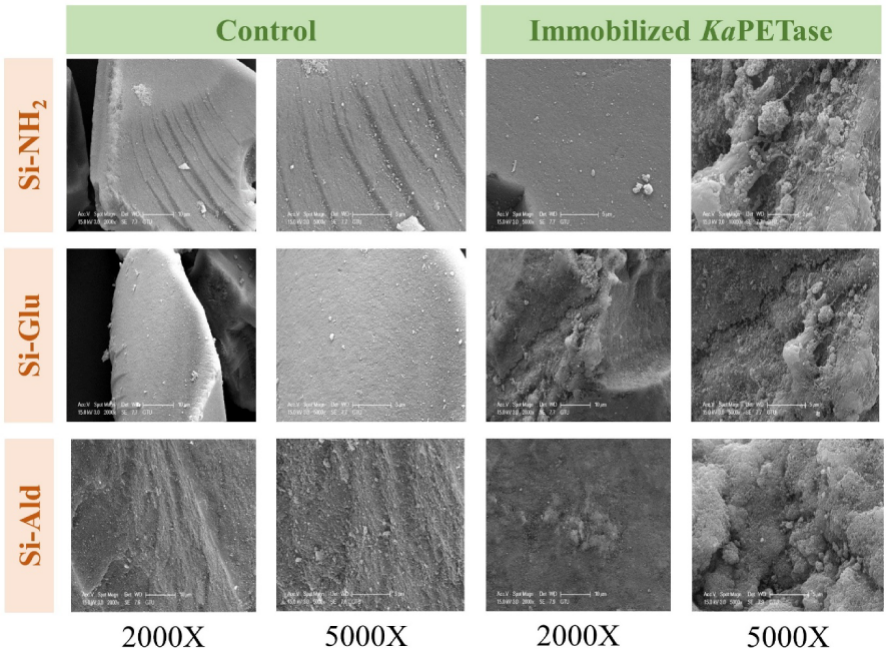
SEM micrographs, acquired at 2000× and 5000× magnifications, illustrating the morphological characteristics of the three functionalized silica supports (Si-NH_2_, Si-Ald, and Si-Glu) before and after the immobilization of *Ka*PETase. The immobilized preparations were generated by incubating 1 g of support with 4 mL of free enzyme solution (0.1 mg/mL) at 25 °C for 2 h, followed by vacuum filtration.

SEM analysis confirmed that all silica support materials contained irregular, sharp-edged particles. Critically, the bare functionalized supports presented visually smooth surfaces. In distinct contrast, the immobilized derivatives Si-NH_2_@*Ka*PETase, Si–Glu@*Ka*PETase, and Si–Ald@*Ka*PETase) exhibited the formation of dense, heterogeneous surface layers. The appearance of this substantial coating on particle surfaces conclusively indicates successful immobilization of *Ka*PETase onto the silica supports. To confirm the immobilization spectroscopically, FTIR spectra of the support materials were recorded before and after *Ka*PETase attachment. All spectra were acquired at room temperature across the range of 4000–500 cm^–1^. The resulting data, which provide direct evidence of successful enzyme immobilization on the functionalized silica supports, are presented and discussed in Figure 2.

**Figure 2:**
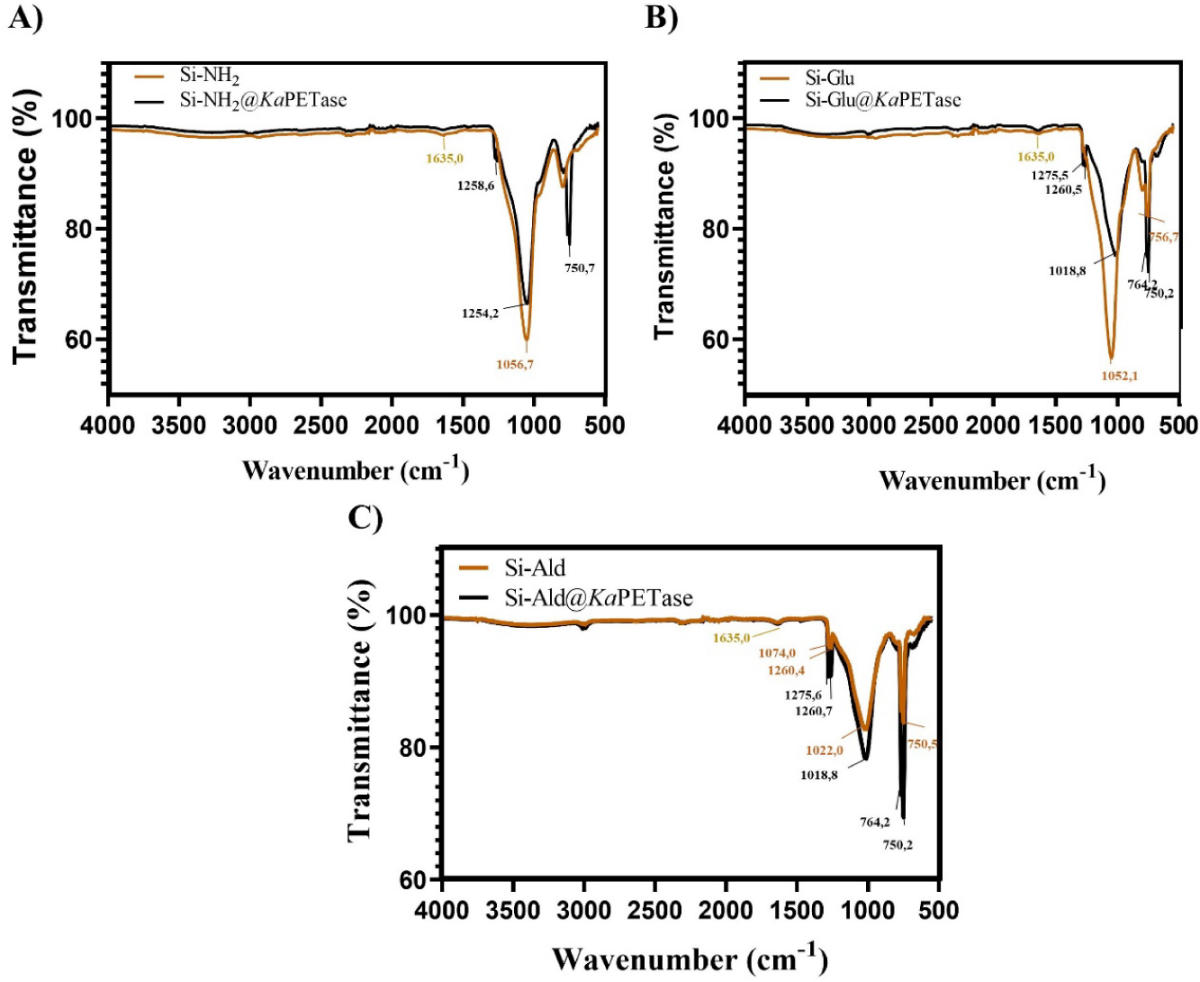
FTIR spectra obtained via the ATR technique, comparing the functionalized silica support materials with their corresponding immobilized *Ka*PETase derivatives. Spectra were recorded at room temperature over the wavenumber range of 4000–500 cm^–1^; A) Si-NH_2_ support and Si-NH_2_@*Ka*PETase, B) Si-Glu support and Si-Glu@*Ka*PETase, **C)** Si-Ald support and Si-Ald@*Ka*PETase.

The FTIR spectra of all silica supports consistently exhibited characteristic broad absorption bands in the 3675–3425 cm^–1^ region, which are assigned to the stretching vibrations of silanol (-Si–OH) OH groups on the support surface.^56,57^ Furthermore, strong absorption bands were confirmed in the 1000–1100 cm^–1^ region, unequivocally corresponding to the asymmetric stretching vibrations of the siloxane (Si–O–Si) backbone of the silica material.^58,59^ Crucially, following enzyme immobilization, the spectra of the Si-NH_2_@*Ka*PETase, Si-Glu@*Ka*PETase, and Si-Ald@*Ka*PETase derivatives displayed the characteristic peaks of an immobilized protein. These include the emergence of band at approximately 1635 cm^–1^ (Amide I), which collectively provide spectroscopic evidence for protein binding to the support surface.^60–62^

To evaluate the thermal stability and quantify the organic loading of the enzyme preparations, TGA was conducted on all bare silica supports and their corresponding immobilized enzyme derivatives. The analysis involved heating the samples from 25 to 800 °C at a rate of 10 °C/min under a nitrogen atmosphere. The resulting thermograms are displayed in Figure 3.

**Figure 3:**
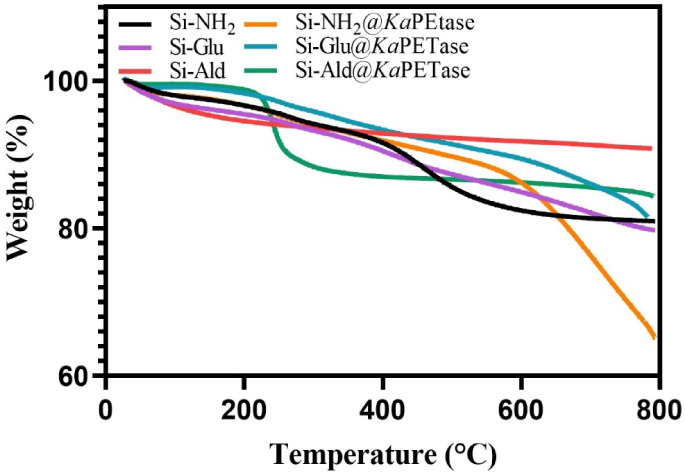
TGA profiles of silica supports and immobilized *Ka*PETase derivatives.

Analysis of the thermograms revealed variations in the inherent thermal stability among the bare support materials. Specifically, the Si-Ald support exhibited maximal thermal stability, suggesting that its surface aldehyde layer is comparatively more robust than the functional groups on the Si-NH_2_ and Si-Glu supports. All samples, including the bare supports and the immobilized derivatives, maintained high structural integrity, retaining approximately 97–99% of their initial weight up to 200 °C. However, in the temperature range of 200–450 °C, the immobilized *Ka*PETase preparations exhibited significantly greater mass loss than their respective bare-support controls. This intensified thermal decomposition in the derivatives is directly attributable to the pyrolysis of the immobilized protein inclusion *Ka*PETase and the organic linker moieties, thereby quantifying the enzyme loading onto each support material.^57,63^ The Si-NH_2_@*Ka*PETase derivative displayed the most substantial mass loss across the degradation temperature profile. This observation is consistent either with the highest protein loading among the preparations or with weaker structural stabilization imparted by its specific immobilization chemistry, which leads to earlier decomposition. In contrast, Si-Ald@*Ka*PETase exhibited a distinct degradation peak in the early thermal stage, centered around 250–300 °C.

### 3.3 Biochemical and Kinetic Characterizations

A comprehensive biochemical characterization of the immobilized *Ka*PETase preparations was subsequently performed. This evaluation included determination of optimal pH and temperature profiles, assessment of long-term thermal stability over a 24-h period, and quantification of operational reusability (Figure 4).

**Figure 4:**
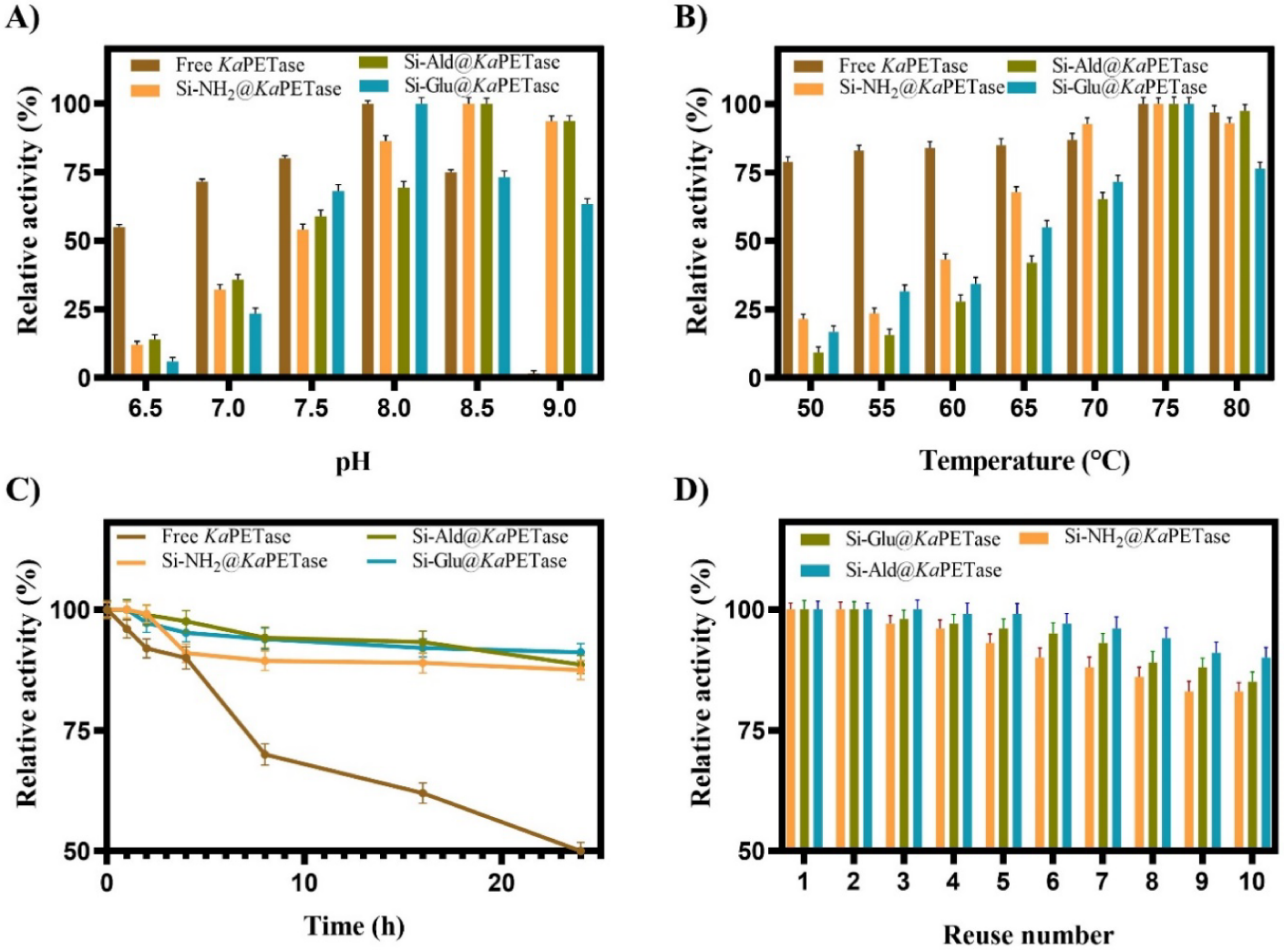
Relative activity results of free and immobilized *Ka*PETase samples against p-NPA. A) Activity change based on pH during the reaction from sodium phosphate buffers (50 mM, pH 6.5–7.0) to Tris-HCl buffers (50 mM, pH 7.5–9.0), B) Activity change based on the temperature exposed throughout the reaction time ranging from 50 to 80 °C, C) Thermal stability of the immobilized *Ka*PETases at 75 °C over 24 h, D) 10 cycles of reusability assay by activity analysis against p-NPA.

The activity of all *Ka*PETase preparations was systematically measured across a pH range of 6.5–9.0 at a constant temperature of 50 °C (Figure 4A). Both free *Ka*PETase and Si-Glu@*Ka*PETase exhibited maximum activity at pH 8.0. In contrast, both Si-NH_2_@*Ka*PETase, and Si-Ald@*Ka*PETase a modest alkaline shift in their pH optima, achieving maximum activity at pH 8.5. In the slightly acidic-to-neutral range (pH 6.5–7.5), free *Ka*PETase maintained a higher relative activity than that of all immobilized preparations. Under alkaline conditions, the immobilized enzymes exhibited higher activity than the free enzyme, whose activity was completely abolished at pH 9.0. The pH-dependent changes particularly the alkaline shift and improved stability may arise from conformational stabilization, in which increased structural rigidity makes the enzyme less sensitive to medium variations, or from microenvironmental effects created by the functionalized supports. Ionic groups on the support (e.g., −NH_3_^+^/COO^–^) can generate local proton gradients, shifting the effective p*K* values of key catalytic residues Similar effects have been reported in other systems, such as the cutinase from *Fusarium solani* (FSC) immobilized onto magnetic genipin-cross-linked chitosan beads, which experienced a pH optimum shift from 8.0 to 9.0.^64^ However, the pH optimum of *Ka*PETase and its ZIF-8 counterparts *Ka*PETase@ZIF-8 and *Ka*PETase@ZIF-8/Glu) remained unchanged at 8.0 in our previous study.^24^ Furthermore, Liu et al.^65^ reported no shift (pH 7.5 for both free and immobilized) upon immobilizing recombinant cutinase onto ET-C@SiO_2_@MNPs, highlighting the complex, support-dependent nature of this phenomenon.

The effect of temperature on the catalytic activity of both free and immobilized *Ka*PETase preparations was investigated over the range of 50 to 80 °C at the respective optimal pH of each biocatalyst (Figure 4B). Notably, all *Ka*PETase preparations (free and immobilized) exhibited a consistent maximum activity at 75 °C. In the lower temperature range of 50–65 °C, the free *Ka*PETase displayed higher relative activity than all immobilized derivatives. This observation is typically attributed to the enhanced conformational flexibility of the free enzyme and the absence of external mass transfer limitations, which often impede immobilized biocatalysts at suboptimal temperatures. This finding aligns with previous studies on *Ka*PETase, where the optimal temperature remained unchanged at 75 °C after immobilization onto ZIF-8 and subsequent cross-linking *Ka*PETase@ZIF-8 and *Ka*PETase@ZIF-8/Glu).^24^ Similarly, the optimal temperature was 70 °C for both free and ET-C@SiO_2_@MNPs immobilized cutinase.^65^ In contrast, the immobilization of FSC onto magnetic genipin-cross-linked chitosan beads resulted in a 5 °C shift in the optimum temperature, increasing from 50 to 55 °C.^64^

The thermal stability of the free and immobilized *Ka*PETase preparations was assessed by monitoring residual activity during incubation at 75 °C (Figure 4C). The free *Ka*PETase exhibited rapid thermal deactivation, retaining only 50% of its initial activity after 24-h incubation. In contrast, the immobilized derivatives demonstrated significantly enhanced stability: Si-NH_2_@*Ka*PETase, Si-Glu@*Ka*PETase, and Si-Ald@*Ka*PETase preserved 87.5, 91.2, and 88.6% of their initial activities, respectively, under identical conditions. Enhanced thermal resilience is attributed to strong linkages formed between the enzyme and the support, which effectively stabilize the intramolecular interactions that maintain the enzyme’s three-dimensional conformation. This stabilization is quantitatively reflected in the *t*_1/2_ values. The *t*_1/2_ of free *Ka*PETase was determined to be 19.2 h, which increased dramatically upon immobilization to 88.4 h for Si-NH_2_@*Ka*PETase, 91.2 h for Si-Glu@*Ka*PETase, and a maximal 97 h for Si-Ald@*Ka*PETase (Table S1). The calculated SF further confirms the protective role of the support, showing that Si-NH_2_@*Ka*PETase, Si-Glu@*Ka*PETase, and Si-Ald@*Ka*PETase are approximately 4.6-, 4.8-, and 5.1-fold more stable than the free enzyme at 75 °C. These results indicate that the functionalized silica provides a rigid scaffold and a protective microenvironment, which together mitigate thermal deactivation. Notably, the short-active group aldehyde support (Si-Ald), achieved through a tighter attachment, provided the highest thermal stability for *Ka*PETase. This performance exceeds that reported for free cutinase, which lost approximately 50% activity after 6 days at 70 °C compared to its ET-C@SiO_2_@MNPs counterpart that retained 84%.^65^ Similarly, other studies have shown that PETase immobilization onto nanostructured Co_3_(PO_4_)_2_ increased residual activity from 17.2% (free) to 82% after 3 h at 45 °C.^23^

The operational reusability of the immobilized *Ka*PETase derivatives, a critical parameter for industrial applicability, was evaluated over 10 sequential batch hydrolysis cycles (Figure 4D). The preparations exhibited excellent retention of catalytic activity, significantly outperforming values reported in the literature. After 10 cycles, *Ka*PETase immobilized on the short-chain aldehyde group (Si-Ald@KaPETase) exhibited higher reuse stability than Si-NH_2_@KaPETase and Si-Glu@KaPETase, with remaining activities of 90%, 83%, and 85% for Si-Ald@KaPETase, Si-NH_2_@KaPETase, and Si-Glu@KaPETase, respectively. Schwaminger et al. immobilized PETase on superparamagnetic iron oxide nanoparticles (PETase@MNPs) and reported that PETase@MNPs retained about 50% of their initial activity after 10 cycles.^25^ Similarly, cutinase immobilized on magnetic nanoparticles maintained 61% of its initial activity after just six cycles.^66^ The exceptional stability observed here confirms that covalent attachment to functionalized silica supports, particularly the Si-Ald preparation effectively creates a biocatalyst sufficiently robust for repeated use in continuous processing.

To evaluate the impact of immobilization on substrate affinity and reaction rate, the apparent kinetic parameters, specifically the Michaelis constant (K_M_) and the maximum reaction rate (*V*_max_), of the free and immobilized *Ka*PETase preparations were determined using p-NPA as the model substrate. The results of this kinetic analysis, including the calculated catalytic efficiency (*k*_cat_/K_M_), are summarized in Table 2.

**Table 2:** Kinetic Parameters of *Ka*PETases against P-NPA under the Optimum pH and Temperature

Biocatalyst	K_M_ (mM)	*V*_max_ (U/mg protein)	*k*_cat/_K_M_ (1/M min)	Catalytic efficiency ratio
Free *Ka*PETase	2.10	16.70	2.6 × 10^5^	-
*Ka*PETase-Si-NH_2_	0.79	16.33	6.8 × 10^5^	2.6
*Ka*PETase-Si-Glu	0.56	15.53	9.2 × 10^5^	3.5
*Ka*PETase-Si-Ald	0.31	12.23	13.0 × 10^5^	5.0

The K_M_ values for the free *Ka*PETase were determined to be 0.79 mM. Upon immobilization, the K_M_ values systematically decreased to 0.56 mM for Si-NH_2_@*Ka*PETase, 0.31 mM for Si-Glu@*Ka*PETase, and the lowest value (best affinity) of 0.20 mM for Si-Ald@*Ka*PETase. This decrease in *K*_M_ suggests an enhanced substrate affinity of the immobilized enzyme toward p-NPA. This enhanced affinity may be attributed either to favorable conformational changes induced during the immobilization process or to substrate partitioning within the microenvironment created by the support material. In parallel, the apparent *V*_max_ values were measured. *V*_max_ was 16.7 U/mg protein for free *Ka*PETase, and remained largely comparable, through slightly reduced, across the immobilized preparations: 16.33 (Si-NH_2_@*Ka*PETase), 15.53 (Si-Glu@*Ka*PETase), and 12.23 U/mg protein (Si-Ald@*Ka*PETase). Despite the marginal reduction in *V*_max_, the significant decrease in *K*_M_ led to a substantial enhancement in catalytic efficiency. The catalytic efficiency ratios (relative to the free enzyme) 2.6-, 3.5-, and 5.0-fold increase in Si-NH_2_@*Ka*PETase, Si-Glu@*Ka*PETase, and Si-Ald@*Ka*PETase, respectively. This demonstrates that all immobilized *Ka*PETase preparations achieved at least a 2.6-fold higher catalytic efficiency than the free enzyme. Furthermore, when compared to our previous work using ZIF-8 as a support,^24^ the functionalized silica-based supports proved more effective at enhancing *Ka*PETase activity Published studies about the characterization of immobilized PETases were listed in Table 3 for the detailed comparison.

**Table 3:** Biochemical and Kinetic Characteristics of the Studied Immobilized Versions of PETases^67^ ^a^

Enzyme	Immobilization support	Optimal pH	Optimal temperature (°C)	Catalytic efficiency	Reusability	Ref.
*Ka*PETase	Si-NH_2_	8.5	75	6.8 × 10^5^ 1/M.min	83 after 10 cycles	This study
*Ka*PETase	Si-Glu	8.0	75	9.2 × 10^5^ 1/M.min	85 after 10 cycles	This study
*Ka*PETase	Si-Ald	8.5	75	13.0 × 10^5^ 1/M.min	90 after 10 cycles	This study
*Ka*PETase	ZIF-8	8.0	75	699.0 1/mM.min	90 after 10 cycles	^24^
*Ka*PETase	ZIF-8/Glu	8.0	75	453.8 1/mM.min	93 after 10 cycles	^24^
*Ideonella sakaiensis* PETase	Co_3_(PO_4_)_2_	7.0	45	ND	70.2% after 10 cycles	^23^
*Ideonella sakaiensis* PETase	Carrier free, Cross-linked enzyme aggregate	7.0	70	14.13 ± 0.20 mL/mg.s	77.7% after 10 cycles	^28^
Recombinant enzyme PETase	Fe_3_O_4_	ND	ND	ND	45–60% after 10 cycles	^25^
Recombinant enzyme PETase	Carrier free, Cross-linked enzyme aggregate	8.0	40	0.001 1/mM.min	0% after 6 cycles	^68^
Recombinant enzyme PETase	Magnetic biochar	7.0	30	ND	ND	^26^
Recombinant enzyme PETase	Mesoporous silica	8.0	40	ND	ND	^27^
Recombinant enzyme PETase	Calcium phosphate nanocrystals	8.0	50	ND	ND	^69^
Recombinant enzyme cutinase	Poly(acrylamide) hydrogel	8.0	70	ND	20–40% after 5 cycles	^70^
Recombinant enzyme cutinase	Kollicoat	8.0	70	ND	50% after 10 cycles	^71^
Recombinant enzyme cutinase	SiO_2_@Fe_3_O_4_	7.5	70	8.14 ± 0.36 ml/mg.s	86% after 11 cycles	^65^
*Fusarium solani* cutinase	SiO_2_@Fe_3_O_4_	ND	ND	ND	ND	^72^
*Aspergillus oryzae* cutinase	ZnO	6.0	ND	ND	ND	^73^
*Rhodococcus sp* cutinase	Divinylbenzene and methyl acrylate resin	9.0	40	ND	ND	^74^
*Pseudozyma jejuensis* cutinase	Fe_3_O_4_	8.0	30–40		55% after 50 cycles	^75^

a ND: not determined.

Among the available studies, no distinct trend in pH optima has been observed, as most enzymes exhibit profiles comparable to those previously reported. In contrast, *Ka*PETase preparations display the highest optimal temperature, which is advantageous for PET degradation given the material’s *T*_g_. Although catalytic efficiency has not been consistently reported across PETase immobilization studies, *Ka*PETase demonstrates activity comparable to other characterized variants. Moreover, immobilized forms of *Ka*PETase show substantial potential, exhibiting high optimal reaction temperatures and enhanced reusability, both of which are critical factors for effective PET degradation and industrial implementation.

### 3.4 Analysis of PC–PET Depolymerization by Immobilized *Ka*PETases

To evaluate the PET decomposition potential of the immobilized *Ka*PETase preparations, both changes in surface morphology and chemical structure of the PET pieces were rigorously investigated using SEM and FTIR. SEM images of PET treated with immobilized *Ka*PETase preparations at 75 °C for 1 day are presented in Figure 5.

**Figure 5:**
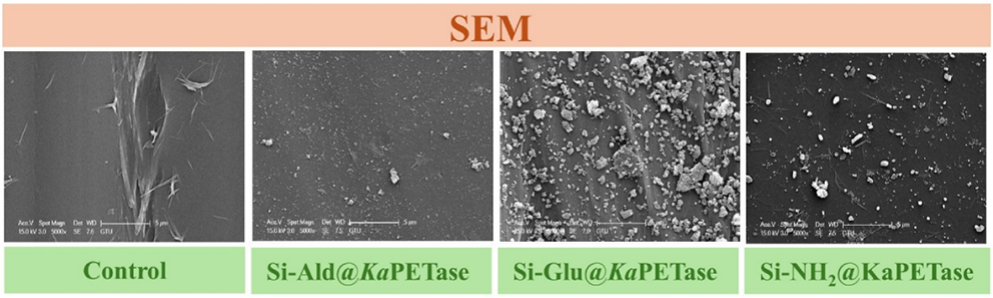
SEM images of PET films incubated with *Ka*PETase preparations at 75 °C for 1 day and incubated at 50 mM sodium phosphate buffer (pH 8.0) as a control.

In the control experiments, no significant changes were detected, whereas small pits and localized surface roughening on the PET substrate were observed during immobilized *Ka*PETase-catalyzed hydrolysis of PET. While the control experiments showed no significant surface alterations, PET substrates exposed to the immobilized *Ka*PETase preparations exhibited distinct formation of small pits and localized surface roughening. These morphological changes are more pronounced, with the appearance of grooves, folds, and pits being more apparent under the 75 °C incubation condition compared to our previous findings.^24^ Similar SEM degradation patterns have been consistently reported across various enzyme-driven PET hydrolysis studies.^23,76,77^ When compared to the previous study,^24^ grooves and folds and pits are more apparent at 75 °C incubation for 1 day. Various applications for enzyme-driven PET hydrolysis showed similar SEM images.^23,76,77^ Moreover, FTIR spectra of immobilized *Ka*PETase-catalyzed PET films provided chemical confirmation of PET cleavage (Figure S2). Specifically, the peak intensity corresponding to the aliphatic −O–CH_2_– group at 1018 cm^–1^ showed a measurable decrease relative to the control treatments. Collectively, these SEM and FTIR results conclusively confirm that the observed PET decomposition was enzyme-driven, with the selective cleavage of ester bonds occurring particularly at the −COO–CH_2_CH_2_O– linkages within the polymer backbone.

X-ray photoelectron spectroscopy (XPS) was employed to elucidate the surface chemical modifications and compositional changes on PC–PET pieces after incubation with immobilized *Ka*PETases. The high-resolution C 1*s* and O 1*s* spectra (Figure 6a–b) of all samples revealed systematic shifts and intensity variations, confirming that successive surface reactions emerged from *Ka*PETase enzyme activity.

**Figure 6:**
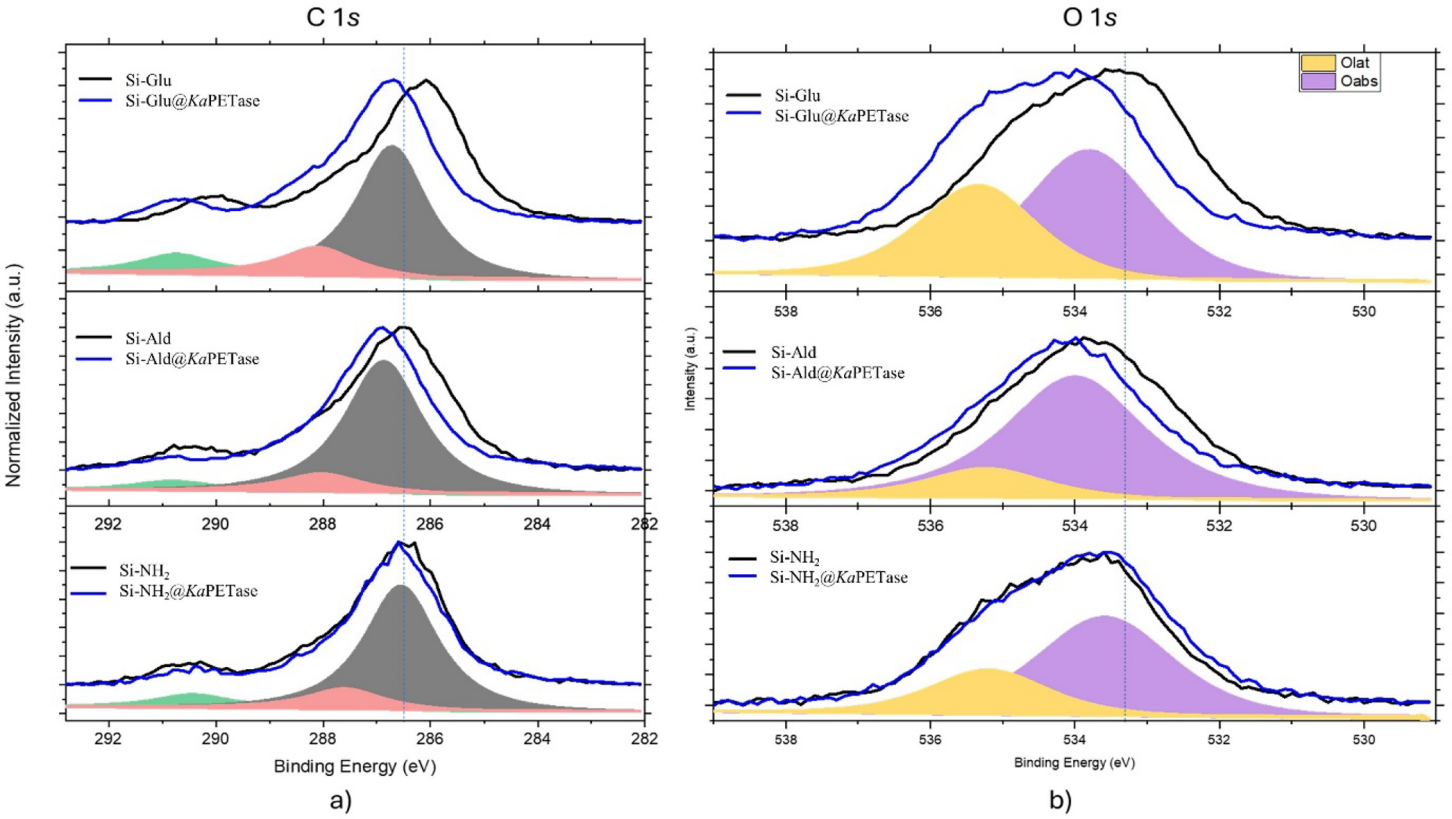
High-resolution C 1*s* and O 1*s* spectra of PC–PET pieces incubated with immobilization support and immobilized *Ka*PETases. The fitted components of each spectrum are displayed. Vertical dashed lines are included to guide the eye.

The C 1*s* spectra were deconvoluted into three principal components: the dominant peak centered around 287 eV, attributed to sp^2^/sp^3^ hybridized carbon (C–C, C=C, or C–H), a component at approximately 288 eV assigned to C–O species, and a minor contribution near 291 eV corresponding to carbonyl (C=O) and other oxygen-rich functionalities.^78^ Following incubation of PC–PET with Si-NH_2_@*Ka*PETase, only minor variations in the C 1*s* spectra were observed on the surface of PC–PET, indicating that the immobilized enzyme did not substantially alter the overall carbon chemistry of the surface. However, the C 1*s* envelope for the PC–PET incubated with Si-Ald@*Ka*PETase showed a spectral shift of approximately 0.5 eV toward higher binding energies, accompanied by an increase in full width at half-maximum (fwhm) and a noticeable reduction in the intensity of the carbonyl-related component. This shift became more pronounced (∼1 eV) for the PC–PET incubated with Si-Glu@*Ka*PETase. The Si-NH_2_@*Ka*PETase, in contrast, displayed only minor spectral changes on PC–PET surface. Such differentiation in spectral responses demonstrates that both Si-Ald@*Ka*PETase and Si-Glu@*Ka*PETase promote effective enzymatic activity on PC–PET.

It was observed greater shifts in C 1s binding energy, increased fwhm, and more pronounced surface chemical modification for PET surfaces incubated by Si-Glu@*Ka*PETase and Si-Ald@*Ka*PETase systems. These findings support enhanced surface interaction and catalytic modification, but not direct proof of covalent enzyme–PET reaction. Therefore, it can be concluded that XPS reveals more pronounced surface chemical modifications and spectral changes, which are consistent with enhanced enzyme–surface interactions rather than direct proof of covalent coupling.^78^ Additionally, almost no detectable nitrogen signal was observed (Figure S3). This is most likely due to washing step of PET compound at the end of incubation with immobilized *Ka*PETase samples. The nitrogen content introduced by the protein layer is below the detection limit of the instrument and/or masked by the dominant silica signal. Therefore, while C 1*s* and O 1*s* spectra were provided to demonstrate changes in surface chemistry, the absence of detectable N 1*s* because of washing step and intrinsic sensitivity limitations of XPS for detecting ultrathin enzime layer on supports.

The depolymerization of PET was quantified using HPLC analysis, which confirmed that the primary degradation products were TPA, and mono­(2-hydroxyethyl) terephthalate (MHET) with a minor quantity of bis­(2-hydroxyethyl) terephthalate (BHET). These results align with the known PET hydrolysis pathway reported in the literature.^79–81^ The retention times of TPA, BHET, and MHET were determined to be 8.90, 19.47, and 26.33 min, respectively (Figure S4). Consistent with observations by Kawai et al.^82^ on the engineered cutinase Cut190, BHET is presumed to be rapidly hydrolyzed into MHET and TPA. Figure 7 illustrates the time-dependent formation of the total degradation products TPA and MHET catalyzed by the free and immobilized *Ka*PETase preparations.

**Figure 7:**
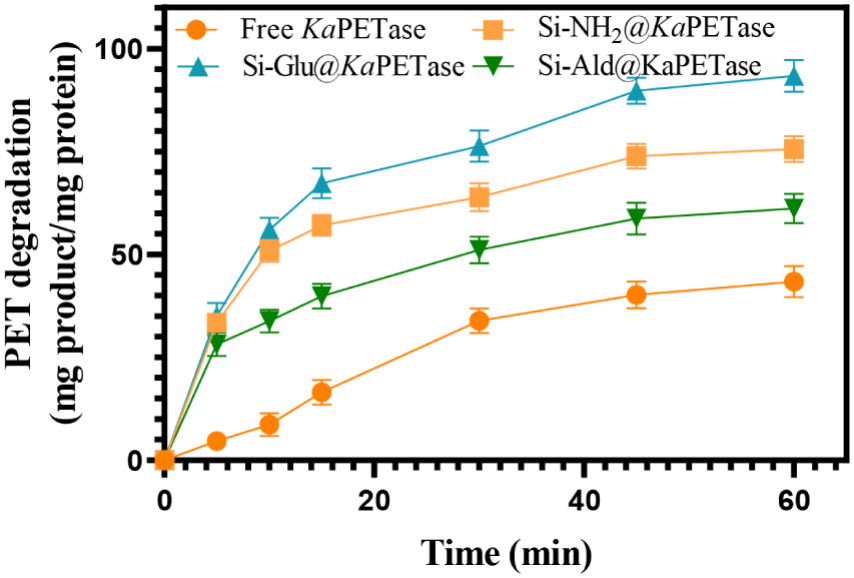
Time-dependent formation of degradation products amount (TPA and MHET) by free and immobilized *Ka*PETase-catalyzed reaction.

Critically, all immobilized *Ka*PETase preparations exhibited substantially higher PET degradation performance than the free enzyme. After 1 h of reaction period, the total formed degradation product per mg of protein was 43.4, 75.6, 93.4, and 61.2 mg for the free *Ka*PETase, Si-NH_2_@*Ka*PETase, Si-Glu@*Ka*PETase, and Si-Ald@*Ka*PETase, respectively. According to these results, Si-NH_2_@*Ka*PETase, Si-Glu@*Ka*PETase, and Si-Ald@*Ka*PETase show 1.75-, 2.15-, and 1.41-fold increase in degradation product, relative to the free enzyme. Although the Si-Ald@*Ka*PETase preparation exhibited the highest catalytic efficiency toward the substrate p-NPA, the Si-Glu@*Ka*PETase achieved the most pronounced XPS binding-energy shifts and the highest TPA formation from the polymeric PET substrate.

Superior performance is likely due to the 3-APTES functionalization followed by glutaraldehyde modification, which may increase the hydrophobicity of the support. This enhanced hydrophobicity, along with the optimal active group length provided by Si-Glu, is hypothesized to improve the accessibility of the *Ka*PETase active site and its interaction with the insoluble polymeric PET substrate.

Our maximal 2.15-fold enhancement is competitive with published literature, such as the 2.5-fold increase in product release achieved by PETase and MHETase coimmobilized on magnetic nanoparticles by Kotnis et al.^83^ Sulaiman et al.^84^ reported that the specific activity of 12 mg/h.mg enzyme for LC-cutinase on PET, identifying TPA as the major product. Furthermore, Hirata et al.^85^ demonstrated a 6-fold increase in total monomer generation using a thermostable mutant cutinase (Cut190∗∗SS) immobilized on SPIONs via a glutaraldehyde active group, highlighting the considerable potential of this strategy. A thermostable mutant cutinase, Cut190∗∗SS, was immobilized on superparamagnetic iron oxide nanoparticles (SPIONs) via a glutaraldehyde active group. The resulting immobilized enzyme (SPIONs-GLU-Cut190∗∗SS) was used to hydrolyze PET at 70 °C. After 48 h of reaction, SPIONs-GLU-Cut190∗∗SS generated 13.45 ± 0.87 μmol of total monomers. Moreover, the immobilized enzyme exhibited a 6-fold increase in monomer generation compared to the free cutinase.

## 4 Conclusion

In this study, *Ka*PETase was successfully immobilized onto functionalized silica supports using aldehyde-based active groups of varying lengths and was confirmed by FTIR and SEM. The immobilized *Ka*PETase preparations exhibited a substantial 5.0-fold increase in catalytic efficiency while maintaining over 87% of their initial activity after 24-h incubation at 75 °C, which is a critical threshold for industrial PET depolymerization. Application tests using PC–PET substrates confirmed the catalytic degradation capability of the immobilized *Ka*PETase biocatalysts as shown by SEM, XPS, FTIR, and HPLC. These findings highlight the promise of functionalized silica as a robust and industrially viable strategy for designing high-performance, reusable biocatalysts crucial for advancing plastic bioremediation efforts.

## Supplementary Material



## Notes

Additional experimental methods, including enzyme immobilization procedures; experimental data, including enzyme stability profiles and PET degradation results analyzed by FTIR, XPS, and HPLC (PDF)
