# Supplementary material for: Design of Functionalized Silica Immobilization of PETase from Kibdelosporangium aridum: Comparison of Glyoxyl and Glutaraldehyde Strategies for PET Depolymerization
Source: ACS Omega. 2026 Apr 20;11(18):27002–14. doi: 10.1021/acsomega.6c00584 (PMC13177217; doi:10.1021/acsomega.6c00584)
Supplement: Supplementary file 1 [file ao6c00584_si_001.pdf]

Design of Functionalized Silica Immobilization of PETase from *Kibdelosporangium aridum*: Comparison of Glyoxyl and Glutaraldehyde Strategies for PET Depolymerization

Buse Çaloğlu Susamaz[a], Mine Nazan Kerimak-Öner[b]*, N.Ece Varan Faki[c], Leyla Colakerol Arslan[d], Deniz Yildirim[c,e], Barış Binay [a,f]*

[a] Gebze Technical University, Faculty of Engineering, Department of Bioengineering, Gebze 41400, Kocaeli, Türkiye

[b] Kocaeli University, İzmit Vocational School, Department of Medicinal and Aromatic Plants, Kartepe, 41285, Kocaeli, Türkiye

[c] Cukurova University, Faculty of Science and Letters, Department of Chemistry, 01330, Adana, Türkiye

[d] Gebze Technical University, Faculty of Science, Department of Physics, Gebze 41400, Kocaeli, Türkiye

[e] Cukurova University, Faculty of Engineering, Department of Chemical Engineering, 01330, Adana, Türkiye

[f] BAUZYME Biotechnology Co., Gebze Technical University Technopark Region, Gebze 41400, Kocaeli, Türkiye

Authors' e-mail information:

Buse Susamaz : caloglubuse@gmail.com

Mine Nazan Kerimak-Öner : mine@kocaeli.edu.tr

N.Ece Varan Faki : evaran@cu.edu.tr

Leyla Colakerol Arslan : lcolakerol@gtu.edu.tr

Deniz Yıldırım : dyildirim@cu.edu.tr

Barış Binay : binay@gtu.edu.tr

***Co-Corresponding authors:**

Prof. Dr. Barış Binay: Department of Bioengineering, Gebze Technical University, Gebze, Kocaeli, Türkiye, Tel: +90 262 605 22 80; e-mail: binay@gtu.edu.tr

Dr. Mine Nazan Kerimak-Öner: Kocaeli University, İzmit Vocational School, Department of Medicinal and Aromatic Plants, Kartepe, 41285, Kocaeli, Türkiye, Tel: +90 262 351 32 86; e-mail: mine@kocaeli.edu.tr

Supplementary Information

Table S1. Thermal stability parameters of free and immobilized KaPETase preparations at 75 °C.

**Biocatalyst**	**k_d (h^{-1})**	**$t_{1/2}$ (h)**	**SF**
Free *Ka*PETase	$36x10^{-3}$	19.2	-
Si-NH_2@*Ka*PETase	$7.8x10^{-3}$	88.4	4.6
Si-Glu@*Ka*PETase	$7.6x10^{-3}$	91.2	4.8
Si-Ald@*Ka*PETase	$7.1x10^{-3}$	97.0	5.1

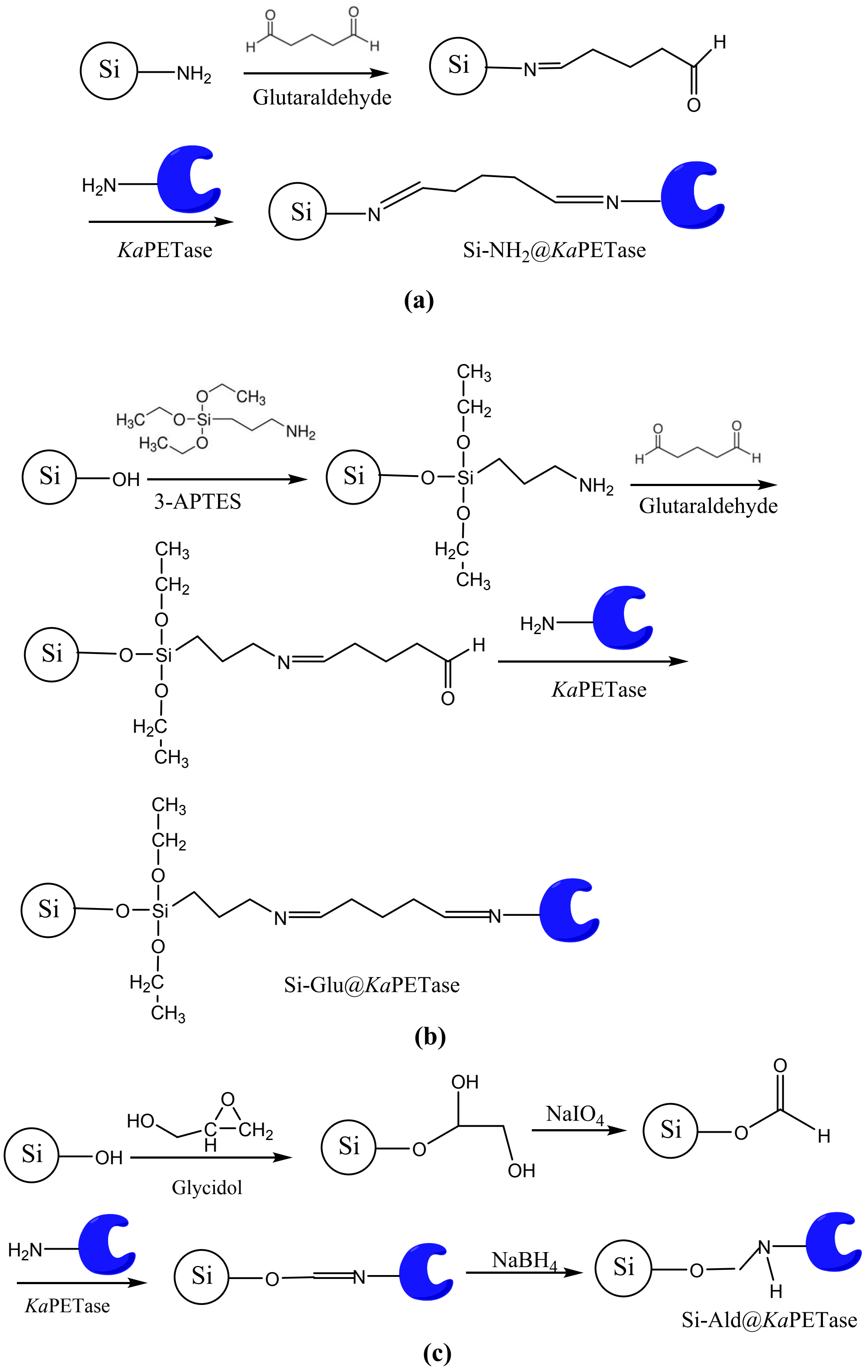

Fig. S1. The preparation steps of the immobilization supports and the immobilization scheme of *Ka*PETase on the supports.

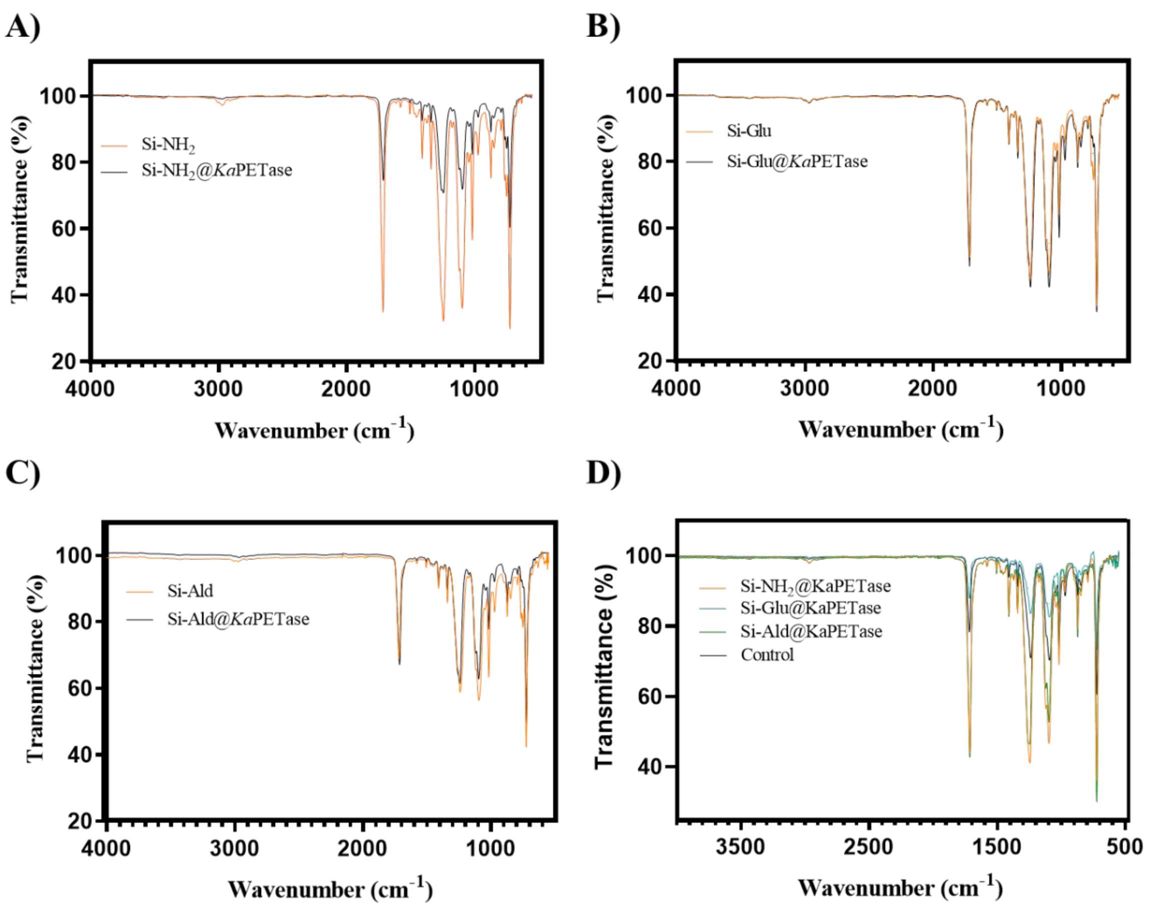

Fig. S2. FTIR images of PET surfaces after incubation with immobilization supports and immobilized *Ka*PETase preparations at 75°C for 1 day (**A**, **B**, **C**) and at 25 °C for 20 days (**D**). Control at D figure expresses incubation of PET piece with 50 mM NaPi pH 8.0 at 25 °C for 20 days.

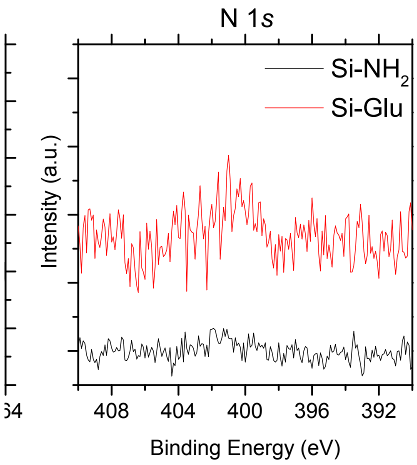

Fig. S3: High-resolution XPS spectra of the (N 1s regions of the for PET incubated with enzyme immobilized with Si-NH_2 and Si-Glu.

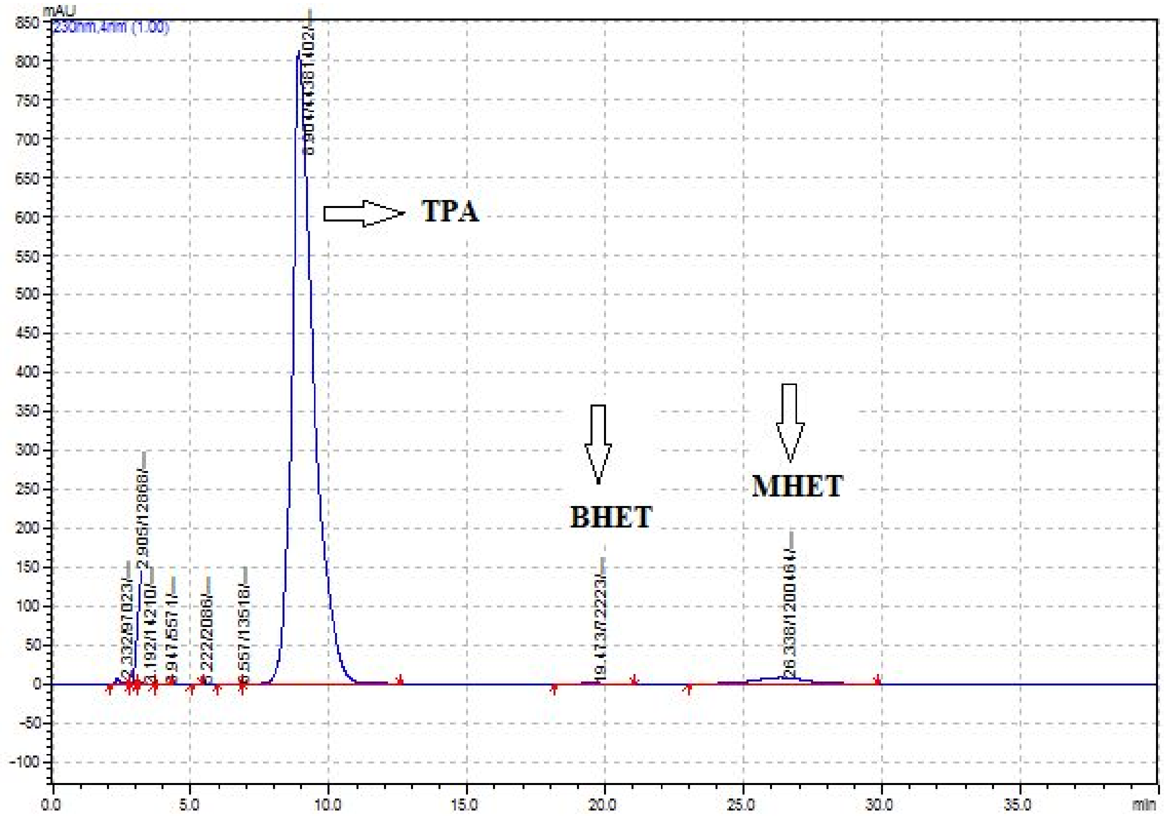

Fig. S4. HPLC chromatogram of solution where PET and Si-Glu@*Ka*PETase incubated.
